# Prosthesis and the engineered imagination: reading augmentation and disability across cultural theory, representation and product design

**DOI:** 10.1136/medhum-2018-011583

**Published:** 2019-03-20

**Authors:** Raymond Holt, Stuart Murray

**Affiliations:** 1 Mechanical Engineering, University of Leeds, Leeds, UK; 2 English, University of Leeds, Leeds, UK

**Keywords:** disability, rehabilitation medicine including disability

## Abstract

This article argues for the value of considering the interaction of literary/cultural studies, disability studies and engineering/design studies in the ongoing development of a critical medical humanities research frame. With a specific focus on prosthesis, but also considerations of embodiment, technology and augmentation as concepts in both cultural/disability theory and engineering/design, we note how the shifting and plastic ideas of ‘the prosthetic’ as used within cultural studies have never been in conversation with scholars who work on prostheses in engineering design or the processes through which such technologies are produced. Additionally, we show that the increased use of systems engineering in the design and construction of prostheses creates fractured ideas of disabled bodies that frequently ignore both the cultural meaning and lived experience of technology use. In design and engineering, prostheses are literal objects, often made to order for a diverse range of clients and produced across different working platforms; in cultural studies, the word creates multiple resonances around both augmented bodies and non-embodied states increasingly understood in terms of assemblage and supplementarity. Working from this, we outline how questions of metaphor, materiality and systems weave through the different disciplines. The article claims that a critical dialogue between the working methods of literary/cultural studies and engineering/design, for all their obvious differences, possesses the potential to create informed and sophisticated accounts of disability embodiment. Our conclusion brings the strands of the enquiry together and points to the merits of engineering the imagination, and imagining engineering, as both a subject and method in future medical humanities research.

Over the last 20 years, a diverse range of scholars working in the humanities have embraced ideas of the ‘augmented’, ‘enhanced’ or ‘prosthetic’ in developing critical approaches to social and cultural phenomena. The terms have aided thinking around not just embodiment and the wider notions of subjectivity and selfhood, but also energised conceptions of history, memory, aesthetics and—conceived of broadly—‘the imagination’, ‘consciousness’ and (especially true of ‘augmented’ of course) ‘reality’. As we will spell out below, from the evocation of cyborgs in contemporary cultural theory to the continued resonance of past atrocities such as slavery and the Holocaust, or from imagined technologised social futures to the permeable boundaries of gender formations, conceptions such as the ‘augmented self’ or ‘prosthetic imagination’ have animated possibilities in thinking of new configurations of subject and place.

It is, however, noticeable that these ideas have not been developed through any particular cooperation with those experts for whom the engineering of augmentation or the design and production of prostheses is a daily undertaking. As is obvious, the manufacture of assistive technologies involves the necessary conceptualisation of the relationship between technology and the body, but those conceptions differ radically from the notions of prosthetic personhood that have become a staple feature of work in—broadly speaking—cultural studies. Our aim here is on one level, then, simple: namely to bring together the questions of theory, metaphor, practice and lived-body experiences that are raised when the distinct disciplinary approaches surrounding ‘augmentation’ or ‘prosthesis’ are made to encounter one another. We will focus on prosthesis specifically because, of all the terms that fall under the loose heading of ‘augmentation’, it is that which produces the most meanings, with ‘prostheses’, ‘prosthesis’ and ‘prosthetic’ all denoting subtly different configurations. In design and engineering, prostheses are literal objects, often made to order for a diverse range of clients; in cultural studies, the word creates multiple resonances around augmentation and enhancement, both of individuals and non-embodied states.

We have also chosen prosthesis because of its clear links to disability, which we define here as a set of experiences and interactions with environments that possess intrinsic value in their own terms and challenge normative and ableist concepts of selfhood and community. During the foundational period of critical studies in the 1990s, seminal work by scholars such as Lennard J Davis and Rosemarie Garland-Thomson focused on ideas of the ‘normal’ and ‘ordinary’, highlighting the often-hidden prejudicial values behind these apparently neutral terms.1 In the last 15 years especially, the subject has turned from these processes of unveiling to express the ways in which different bodies challenge assumptions about normalcy, utility and the perception of integrated selfhood. As Michael Davidson observes of the relationship between disability and aesthetics, ‘Disability aesthetics foregrounds the extent to which the body becomes thinkable when its totality can no longer be taken for granted, when the social meanings attached to sensory and cognitive values cannot be assumed’.2 In figuring prosthetics, ideas of the ‘totality’ of the body and the ‘social meanings’ that accrue from these are thrown into sharp relief. Owning or using prostheses signals disability to a majority non-disabled culture, but under whose terms and how? And does it also signal technology in the same way? Whether the perceived absence of a limb is compensated for by an understood presence of a designed and produced alternative is an issue more complex than it might appear. Not all prostheses are replacements, for example (the limb may never have existed in the first place), and there are many different types of design and technology. Staying alive to Davidson’s reminder about what ‘cannot be assumed’, we are guided here by the knowledge that disability acts as a refractive lens through which to read the interrelationship between the body and the technology that interacts with it.

In developing this thinking, we wish to argue for the many benefits that accrue from processes of an interdisciplinary dialogue between cultural/literary studies and engineering, and chart how these then provoke potentially innovative categories of health, disability and the body. In the context of an interdisciplinary critical medical humanities, we want to ask: what might a research space look like that makes room for the combined engineered/imagined body? How can the designed/produced object, and the theorised/abstracted/represented self, extend what we can say about grounded and conceptualised selfhood? How might the *details* of each discipline, whether (for example) the vital technical specifics of engineering or the picking apart of language or trope in cultural studies, allow for an understanding of differing methodologies in which augmentation or enhancement might be framed? We want to structure our answers to these questions in three parts: first, through an exploration of the manner in which cultural theory has taken up the idea of the prosthetised body; second, reading those engineering and design methodologies that illustrate the processes of producing prosthetic technologies; and finally, outlining the outcomes from these interdisciplinary encounters that can inform the multistrand critical approaches that increasingly characterise cultural research undertaken on health and disability. While our examinations have to be necessarily limited, we believe that the shape of the arguments we make points to broader possibilities of integrating humanities and engineering scholarship within the developing discipline of the medical humanities.

## Cultural theory, prosthesis and disability

Augmentation, amputation and replacement have histories as long as congenital physical difference has existed and cultures have intervened in embodiment and health. And across time periods and cultures metaphor has worked to give such difference and interventions meaning. In his history of automata in the Western imagination, Minsoo Kang notes that from Classical times ideas about the technological connections between human and non-human have always ‘functioned as conceptual chameleon[s]’ and as mechanisms through ‘which Western culture has pondered the very nature and boundaries of humanity’.3 Scholarship on disability over the last 40 years has focused on such ideas of conceptual meaning and boundaries. While social model theories located technology as central to disabling environments that create disability as a ‘problem’ (eg, architecture that fails to take into account issues of access or the development of complex online systems that exclude those with specific disabilities), the rise of cultural disability studies as an academic subject area from the 1990s onwards revised the idea that disability, read simply in terms of absence, lack or loss, was corrected by technological intervention. The disabled body, as James Porter notes, operates within a classic double bind, appearing as ‘*too much* a body, too real, too corporeal’, but because of this difference also *‘* *too lit* *tle* a body: a body that is deficiently itself, not quite a body in the full sense of the word, *not real enough’*.4 Such an ambivalence informs much work on the interaction between disability and technology: new developments in smart prostheses, neural implants, exoskeletons or cosmetic augmentation have prompted research that reads the complex status of the body’s boundaries as it meets technological intervention in terms of conceptions of selfhood, interactions with community and status as metaphor.5

The particular contemporary use of ‘prosthetic’ as a critical theoretical term, however, has established specific terms for such metaphors. In 2004, Alison Landsberg entitled her monograph exploring the ways in which ideas of remembrance are articulated through American popular culture, *Prosthetic Memory*. For Landsberg, the meaning of prosthesis here was one of joining, a process of ‘experience’, through which ‘the person sutures himself or herself into a larger history’ that she or he would not personally have known (such as war or conflict, for example). As such, she asserts, ‘prosthetic memory creates the conditions for ethical thinking precisely by encouraging people to feel connected to, while recognizing the alterity of, the ‘other’’.6 It is in this connection to ‘the other’ made here that we might identify one of the central ways prosthesis works in its current cultural formations; namely as an outlining of a projection, with the extended self or selves linking to others through processes of ‘thinking’ or contemplation. In this guise, the ‘prosthetic’ becomes a trope that allows for a critical movement ‘beyond’ static modes of evaluation, particularly dualisms or binaries. As Margrit Shildrick has noted in her work on prostheses and disability, ‘whereas traditional understandings have centred on their utility for a subject experiencing some form of lack or inability, more contemporary approaches stress that prostheses speak to the mode of supplementation’.7 These notions of supplementarity conceive of new critical spaces in which both bodies and subjects might be understood.

Within this logic, prosthetic ‘aesthetics’, ‘imaginaries’ or ‘consciousness’ stress links between and across states in which conventional subject knowledge is extended and developed. In her 2014 book *Phantom Limb: Amputation, Embodiment, and Prosthetic Technology*, Cassandra Crawford explores how, in her terms, ‘innovations in prosthetic science have also transformed the prosthetic imaginary’, noting that ‘bodies have been mediated by prosthetization’ in the production of ‘phantoms’ and ‘embodied ghosts’. ‘Prosthetization is not simply or straightforwardly done to bodies’, Crawford asserts, ‘it is always a relational process of technologization-in-the-making’.8 Also with images in mind, Maria Neicu uses the term ‘Prosthetics Imagery’ to frame her exploration of the ‘identity of enhanced bodies’ and otherness in examples of what she calls ‘Posthumanist Bioart’: artworks and exhibitions in which creativity, ethics, objects and spectatorship are all challenged by ‘an essential rethinking of ourselves and others’.9 In both these examples, an idea of prostheses allows for—even creates—a shuttling between critical perspectives on technology, bodies, imagination and selves.

It is imperative to stress, however, that these usages function, whether knowingly or not, through specific strategic conceptions of disability. Largely unconsciously, the formations of ‘prosthetics’ cited above mobilise disability as a reservoir of fluid content and multiple embodied possibilities, from which any number of ideas might be selected. In response, many of those suspicious of the ways in which the ‘augmented’, ‘enhanced’ or ‘prosthetic’ is articulated in cultural theorising focus particularly on the erasure of *actual* disability meanings produced through such approaches. Much of this scepticism revolves around the understanding of the place of embodiment in this theorising, with concerns that the frequent ignoring of bodily meaning erases the lived nature of disability. In substantial articles, both Vivian Sobchack and Sarah S Jain have addressed what Sobchack calls the ‘seductive’ nature of prosthesis and augmentation understood as metaphors or tropes. ‘The scandal of the metaphor’, Sobchack notes, ‘is that it has become a fetishized and ‘unfleshed-out’ catchword that functions vaguely as the ungrounded and ‘floating signifier’ for a broad and variegated cultural discourse on technoculture that includes little of these prosthetic realities’.10 Jain is similarly concerned about the ways in which the ‘proliferation’ of the term ‘has overburdened it’. As she notes, ‘theories themselves can be, after all, both enabling and wounding’. Specifically, Jain explores the structures through which ‘the disavowal and simultaneous objectification of the disabled body is at stake in the term ‘prosthesis’’, processes often lost in the formation of the words as free-floating category.11

Embodiment is central to the tensions that exist between readings of prosthesis as metaphor or materiality (the latter is often experiential of course, and not simply ‘read’). As Sherryl Vint has shown in her study of technology and subjectivity in contemporary science fiction, ‘Western culture remains attached to a concept of self as disembodied, a concept of self that has important consequences for how we understand the relationship between humans and the rest of the material world’. What Vint terms ‘the (impossible) desire to escape the vicissitudes of the body and occupy the place of self-mastery’ stems from the heritage of Cartesian dualism, where an asserted power of the mind to transcend the (usually degraded) body has been played out in multiple forms.12 Vint has no particular focus on prosthesis or disability in her book, but her insight helps frame the methods through which the ideas that surround prosthesis operate.

Where, then, is the body in the configurations of ‘prosthesis’ outlined above? It appears, both usefully and problematically, to be wherever one might look for it: situated yet diffuse, present and absent at the same time. Possibly a way to locate it more meaningfully is not to keep concentrating on the flesh of the body as a whole, but rather to focus on the ‘the prosthetic’ itself—the object that emerges through processes of conception, vision and design to production and use. How might we read the body, in all its different forms, through the specifics of engineering?

## Engineering systems and networks

Within engineering, ‘design’ is a broad term that covers a spectrum of activities involved in developing any artefact, from the largely creative (‘art and design’) to the predominantly analytical (‘engineering design’). There has long been a debate about whether design is a ‘science of the artificial’13 amenable to laws that will always lead to good design, or a more intuitive process of ‘reflection-in-action’,14 where designs evolve through the application of experience and intuition. In practice, as demonstrated by Louis Bucciarelli’s ethnographic study of engineering design, these approaches are not mutually exclusive, and different stages of the design process, different products or even different parts of a product may require more of one approach or the other.15 Irrespective of where on this spectrum a given design falls, all designs are concerned with the artificial: the outcome of a design process is never ‘natural’ or purely accidental. While these terms and the ideas they generate may influence a design process, the form a given artefact takes is ultimately determined by decisions taken in its development. In terms of the physical body that presents itself to the designer and engineer then, it is an origin and source but also a constraint, the starting point for the processes of production but also, inevitably, the source of their parameters.

One of Bucciarelli’s key observations is that engineering design progresses not as a series of predefined steps, but as a socially negotiated process in which different individuals frame problems and apply methods with which they are familiar in order to address them. In place of a fixation on ‘the object as a thing in itself’ (a prosthetic limb, for example), he stresses ideas of vision, harmony and ‘a cultural matrix’. Where it might be expected that an engineer will hone in on the fine details of a design, Bucciarelli stresses the need to ‘unfocus’, and to then ‘start with a broad canvas, hold suspect the categories and relations we unconsciously accept today, and seek […] evidence of relations in the making and using’ of engineered products.16 As Graham Pullin notes in his foundational study *design meets disability*, this can exacerbate the tension between the creative and the analytical approaches to design. Pullin notes that the development of medical devices, including prosthetics and other assistive technologies, is dominated by engineering designers, who tend to adopt a technical perspective in which the product is purely functional, intended to perform given tasks within given constraints:

‘Traditionally, design for disability has paid more attention to the clinical than the cultural diversity within any group. The same prostheses, wheelchair, and communication devices are often offered to people with a particular disability, whether they are seventeen or seventy years old, and regardless of their attitudes, towards their disability or otherwise’.

In place of this, Pullin echoes Bucciarelli’s call for a ‘broad canvas’ approach, including more art-school-trained designers and those with disabilities:

‘The design issues around disability are underexplored […] and demand and deserve far more radical approaches […] What is needed is truly interdisciplinary design thinking, combining and blurring design craft with engineering brilliance, therapeutic excellence and the broadest experiences of disabled people’.17

Here, the stress on a greater holistic view of users and contexts for product design are key; and when Bucciarelli observes that ‘the artefact as object can live again. It can become a nexus or icon of social discourse or exchange […] There are other object worlds within which the artefact can be seen and used in different ways. Deconstruction and bricolage are always possible’, he is using language that suggests an explicit connection between engineering design processes and the kind of fluid conception of prostheses as trope or metaphor outlined in our first section.18 This specific citation of deconstruction and bricolage, it might also be noted, evidences a knowledge of the poststructuralist critical methods that underpin much of the way in which ‘prosthetics’ have come to be discussed in contemporary cultural criticism.

Bucciarelli and Pullin’s interventions serve to remind that the days of the individual artisan both designing and making a product for a specific user are for the most part gone. Much product development—including prosthetics—is undertaken by teams with a range of skills and corresponding views of the goal to be achieved. As systems have become more complex, systems engineering has become a common approach to coordinate the efforts of different individuals within a development team.19 This entails following a ‘divide and conquer, combine and rule approach’, dividing the product into subsystems, each with its own goal and constraints and allowing a given individual or team to focus on their subsystem, without reference to those of others. Within this subdivided nature of any complex engineering project, different individuals may well deal with different aspects of the body. For example, the designer of a socket to fit a prosthetic is concerned with the shape and structure of the body; the designer of software to interpret electromyography signals will be more concerned with the signals generated by myoelectric activities and the subcutaneous positioning of nerves and muscle fibres; and the designers of the joints and actuation of a prosthetic will be interested in the propagation of forces through the body, and the strain this places on different parts of the body. As a recent Royal Academy of Engineering report into the work of systems engineering puts it:

‘Systems that work do not just happen – they have to be planned, designed and built. There are many ways of formalising what is half an art and half a process; […] successive stages of partitioning, so that the task is broken down into manageable chunks […] successive stages of integration, bringing the chunks together to create the working system’.20

While the report describes a clear focus on the need for breaking down and partitioning, it is worth noting that it is here seen to be both a process and an art. The design and production of the ‘manageable chunks’ still require the vision that reassembles them into a finished system.21

A systems perspective raises issues of networks and connections, with the product being developed itself part of a larger system that interacts with other systems: users and other individuals with whom the product comes into contact; other products to which it connects; and the general surrounding environment. A key decision being taken in developing a product then is not just its internal architecture—how it breaks down into subsystems—but also the scope of the systems’ boundaries: what is seen as acceptable to be altered and what is taken as an external constraint. This can allow for the kind of interdisciplinarity Bucciarelli and Pullin advocate, where boundaries are flexible and to be transgressed; but equally this idea of systems might *require* separation and differentiation to incorporate the necessary specialisms involved in production.

The structures of engineering systems are particularly relevant when it comes to working through engaging with questions of embodiment. Traditionally, the body—understood as the user or users—has been configured as something external to the product: something to be accommodated and adapted around. The product (a prosthetic limb, for example) may be attached to this body, or need to accommodate it in some form, but it is generally produced through a conception of empirical material that corresponds with use value, for example a set of anthropometric measurements (lengths of typical body parts or strengths of different grips).22 Echoing the observations of Bucciarelli and Pullin, however, there have been recent critical reconfigurations that take a more holistic view of users and the systems they suggest (incorporating individual aspirations and social relationships for example), and treating them as more than just a collection of measurements that allow for an object to perform a task.23 In addition, the body is not *always* seen as existing outside the scope of design. Tissue engineering and the design of prosthetics such as replacement hip and knee joints include the alteration of body tissue, while developments in surgical technologies now allow for alterations to bodies that enable nerves to control a prosthetic limb.24

The body, then, takes a range of forms in engineering design. It can be outlined using anthropometric data denoting typical dimensions or capabilities, or it can be framed within mathematical models that capture the biomechanics of human movement or the propagation of force through bone and other tissue, with corresponding stresses, strains and deformations. Such representations are noticeably atomistic rather than holistic: they have very specific purposes and roles in the design process, intended to address very particular questions, which must then be reintegrated at a higher level into the design as a whole. This is inherent to the systems approach often adopted in engineering; the complexity of systems means that it is difficult/impossible for any single person or method to consider every aspect of a design simultaneously. As a result, engineering design rarely thinks of the body as a whole unit or entity; it is always subject in some way to subdivisions that necessitate the atomistic ‘divide and conquer’ approach mentioned previously, into which the various tools and specialisms required to produce the product are designed to fit.

To address this, ‘personas’ are often used in product development: artificial characters who are given a backstory, including details of preferences, family, job, hobbies or aspirations, to think through how they might respond to a given product.25 Such ‘characterization’ offsets a simpler process in which product users are viewed as collections of data that can be modelled objectively. There are, of course, controversies over this: are such ‘characters’ really representative of the intended user? Are they fabricated to suit the prejudices of developers? At what point are choices made as to the details of backstories? What, for example, is being assumed about what an individual might *want* when they are having a prosthetic hand-fitted? Seen through a critical disability lens, it is intriguing to juxtapose the fragmented sense of systems engineering with this corresponding desire to render an idea of a ‘whole’ user. It is a formation distinctly different from the porous or elastic body configured in humanities criticism, one where an idea of ‘the prosthetic’ might be made to connect to a totalising system such as ‘memory’. It also appears to invite a disability reading: if the manufacture of a prosthetic limb takes place within contexts that stress division and separation, it is hard to see how the resulting product can be seen as being integral to an individual’s experience *as* a person with disabilities.

Such perspectives may well be correct, but we feel that there is a danger of misrepresenting the critical methodologies and working practices of design and engineering if they are only read in such terms. For example, it is possible to see congruity rather than discrepancy in some of the differences outlined above. The characterisation of porous embodiment can be seen to be in line with elements of systems thinking; the precise boundary of what is to be engineered can be drawn in different places; an amputation and the prosthetic limb constructed as a response can accommodate more, or less, of the living tissue under the skin and non-living matter beyond it. Seen in such a light, Donna Haraway’s famous provocation ‘why should our bodies end at the skin?’ has a parallel with certain modes of engineering decision-making. What is sometimes referred to as the ‘fuzzy front end’ of engineering—the early stages of determining what it is that is being engineered and what it is hoped will be achieved by it—involves judgements necessarily conceived of in terms of flexibility.26 In the case of prosthetics for example, decisions might be made as to whether the goal is to ‘restore’ a limb following an amputation, or to re-establish specific functions, the latter process one that might be achieved by means other than 'replacement'. These two possible eventualities are not the same, and the body that falls under discussion as a result of choices made in such circumstances is one that cannot be said to be singular. It is impacted differently and with different results. This is, we suggest, an example of where engineering design methods address the (flexible) boundaries of embodiment in ways that have parallels (suggestive at least) with critical cultural and disability studies.

Equally, designing in relation to disability cannot avoid its insertion into narrative or engagement with metaphor. Is the design of a prosthetic limb, conceived of within a context of improvement for example, a part of a narrative in which technology makes ‘our’ lives better? What ideas of function or expression are involved when designing a prosthetic hand, given that the labour any hand produces ranges from the utility of prehension to the emotion inherent in a pointed finger or clenched fist? These thoughts are unavoidable interventions, not only into the specifics of any body, design or individual, but also the complex historical trajectories that make up the ways in which technology and health are juxtaposed. Our contention is that we are better placed to address the complexities of these points of view when we bring the equally complex critical approaches of each discipline into dialogue with one another.

## Combining materiality and metaphor: embodied and ambiguous

Our explorations across the processes of engineering and imagining prosthesis show that it is the combinations of materiality and metaphor, of systems and selves, that form the most productive critical spaces in which the terms can flourish. In a wonderfully illustrative phrase, Sobchack concludes that her own prosthetic limb is ‘*dynamic and situated* but also *ambiguous and graded’*, a personal observation that is also a critical/theoretical intervention stressing *both* the embodied nature of wearing a prosthetic limb as well as its social meanings.27 Here, there is no hierarchy between prosthesis design, disability experience and cultural narrative; none of the three acts as a precursor to the others. Rather, the relationships function through being *networked* in everyday usage; the limb is a manufactured object, almost certainly made within the frame of the kinds of decision-making outlined above, but it is also a bodily attachment, producer of experiences, historical artefact and object of stare.

In terms of an explicit connection between imagination and engineering, Manuela Rossini has coined the term ‘imagineering’ for the ways in which texts conceptualise bodies within networks that anticipate the future, with her deliberate collision of words suggesting an interaction between fictional approaches and a conception of engineering practice. When she observes what she terms the ‘double movement’ of technology and the literary, she makes an important point, namely that ‘literature does not merely react to technological development and offer ethical guidance’. Rather the process is one of greater equality: ‘the technological potential will affect the way the human body/subject is defined but these new meanings (produced in texts and images) will influence, if not our actual use and even deployment of them, our handling of technologies’.28 To ‘imagineer’ then might be to deploy the various versions of design and expression for which we argue here.

However, while Rossini is deft in her analyses of cultural theory and fictional texts, her critical approach does not break down ‘technology’ in any way, leaving nothing that might allow for a focus on how actual engineering methods can contribute to a critical interdisciplinary conception of ‘prosthesis’. There are no specifics about the *work* of design or production in her asserted ‘double meaning’, no account of the complexity inherent when conceiving of production design; the detail only comes from one side. To observe this is to register those moments when cultural criticism, always piratical in its methods, lays claim to terminology and (broadly conceived) ideas from disciplines beyond its own but displays no real care (or courtesy) towards those other subjects. For Rossini, ‘engineering’ is just a set of generalisations attached to a word.

Such omission is significant, because any engineered or designed object is not merely a collection of physical matter, but always at some level the result of an intent.29 The ‘success’ of a design is determined by the match between its physical nature, its environment and the intention of its designers. Or, it might be better to say, intentions, since individual (and teams of) designers and engineers may have different views about the purpose of the system they are developing, which may in turn be different from the views of the end user. If an engineer fails to appreciate the way their choices will interact with the complex network of relationships that exist for a given user, then the outcomes of choices may be very different from those imagined when designing the prosthesis. This process is what makes the imagination so important in engineering, not merely in terms of creative synthesis (imagining new ways of solving problems), but also in imagining how those choices will play out in practice.

Working from these observations, it is our contention that the meeting of engineering and humanities methodologies can offer a productive platform for future thinking on both made objects and the ways in which they are constructed in terms of cultural meaning. Developments in assistive technologies for those with disabilities, artificial intelligence (AI) and robotics are all future moments that can benefit from interactions that are produced when we engineer the imagination in the ways we explore here. The evolving complexity of humanoid care robots, for example, will be better served by an understanding of the complex philosophical and social meanings of care that accompany ageing and disability. ‘Future’ is not often an idea that is productively associated with disability of course. Alison Kafer has observed—critically—that, for many, ‘a ‘good’ future naturally and obviously depends upon the eradication of disability’, and that ‘that this kind of ‘elsewhere’, one without disability, is one ‘we’ all want’.30 But an interaction between engineering and critical disability studies can be exactly the kind of platform that *does* conceive of disability futures in positive terms. Understanding technologies in the ways that we hope are suggested by our intervention here is a process that can both celebrate engineering expertise and respect the situated expertise it receives in the lives of individuals with disabilities.

There is, of course, nothing new in examining the relationships of bodies with and in technology. As Despina Kakoudaki observes in her study *Anatomy of a Robot*, intersections between bodies, technologies and conceptions of health predate the modern era by many centuries. There is a danger, Kakoudaki asserts, of reading such moments of engineering ‘without sufficient regard to historical context or textual provenance’, and her work stresses the need to emphasise the historical and cultural narratives of technological advancement. Advancing this argument, Kakoudaki notes that the designed and produced object ‘holds little cultural sway without the literary and cultural context that would make its performance meaningful or attractive’. In contemporary culture, she argues, such performances ‘inform a host of cultural domains and debates, participating in a dense web of interactions between function and reality’.31 We take such observations on board, and so recognise that it is in the history and narratives of the present that we will find the configurations of contemporary technology, health and disability that help to form notions of self and community.

But, we assert, it is not in these areas alone that meaning is located. The converse to Kakoudaki’s argument is true, and we need to admit that we cannot explain cultural stories of bodies without understanding the role of technological origins and function. As Bucciarelli notes: ‘Technology is object and technique, but object and technique *inside* culture […] Technology as artefact, as system, tool productivity, efficiency – yes; technology as metaphor, as process, as values we live by and in as well’.32 The details in new designs of bespoke prosthetic limbs, with ultrasound technology, electronically measured skin tone or three-dimensional printing, allow for more sophisticated conceptions of cultural embodiment through ever-evolving iterations of the ‘artificial’, while the idea of space inherent within AI shapes reconfigurations of social formations as networks and assemblages and the critical meanings we make of them. As an example of this latter point, how might we read contemporary cultural theory on the body *through* the shapes that current engineering software use to process data? Or read the decision trees central to machine learning’s processing of data next to the ‘arboreal/rhizomatic’ distinction pivotal in the thinking of Gilles Deleuze and Felix Guattari, theory that underpins much contemporary thought on posthuman bodies? Writing on the boundaries of embodiment, Shildrick stresses the value of thinking through the disabled body using the ‘Deleuzian reading of connectivity’ and especially *‘* *the* *term* *assemblage’*. Advancing her argument, Shildrick notes that Deleuze and Guattari’s ‘characterization of assemblages’ was in part enabled through a process of recognising their status of ‘desiring machines’ within a platform of technology.33 Shildrick does not develop a specific focus on the detailed workings of technology as she explores the meaning of prosthetics and supplementarity, but it is perfectly possible to see ways in which critical engineering methodologies could add to her theoretical approaches—matching her sense of ‘re-imagining’ prostheses—towards the boundaries and limits of the body. What, we want to ask, might the consequences be of a decision to orient criticism that way?

Work undertaken in the critical medical humanities has developed key parameters of entanglement and risk that, we feel, map on to the kinds of intersections we are suggesting.34 Equally, a productive version of the latest critical thinking in posthumanism could also function to bring together and make a home for these disparate ideas. In terms that have obvious relevance to the use of prostheses, Pramod Nayar has articulated that ‘critical posthumanism calls attention to the ways in which the machine and the human and other life forms are now more or less seamlessly articulated, mutually dependent and co-evolving’, and that—to focus on the specifics of embodiment—the contemporary body is best understood as being ‘less as a bounded entity than as a network or assemblage, evolving with technology and then environment’.35 (Nayar’s metaphors are mirrored in Bucciarelli’s description of technology as being ‘integral, constitutive of a seamless web, but transcending science and its logic […]. It weaves through our everyday thinking, educating, family raising, churchgoing, leisure and labour’.) In a similar vein, Rosi Braidotti argues that the posthuman subject exists within a frame ‘of multiple belongings, as a relational subject constituted in and by multiplicity […] a subject that works across differences and is also internally differentiated, but still grounded and accountable’. She continues: ‘Posthuman subjectivity expresses an embodied and embedded and hence partial form of accountability, based on a strong sense of collectively, relationality and hence community building’.36 Such terminology can easily be seen to fit with the critical mobility surrounding the body, disability or ‘prostheses’ outlined in our first section here, but we want to argue that Nayar and Braidotti’s terms—‘mutually dependent’, ‘co-evolving’, ‘grounded’, ‘accountable’ and ‘community’—are words and ideas that also drive thinking in design and engineering, whether the mutually dependent evolution of disability design, grounded nature of production, accountability towards clients or community of users.

Ultimately such terms return to the body, the technologies we bring to them and how we make meaning of this interaction. They form the scaffold for a critical practice that can be about both the theorised and situated nature of health and disability. If, as David Mitchell and Sharon Snyder assert in their seminal disability studies work *Narrative Prosthesis: Disability and the Dependencies of Discourse*, ‘the prostheticized body is the rule, not the exception’ in a contemporary culture criss-crossed by connections of technology and subjectivity, then it should be clear that criticism requires the perspectives of both the humanities and sciences in reading the entanglements that are created as a result.37 Given that individuals from all the various communities we have mentioned here are deeply invested in creating positive disability experiences, we see every reason why their work—in all its diversity—should be read in a productive combination that imagines engineers even as it engineers the imagination.

## Notes

1. See Lennard J Davis (1995), *Enforcing Normalcy: Disability, Deafness and the Body* (London and New York: Verso), and Rosemarie Garland-Thomson, 1997, *Extraordinary Bodies: Figuring Physical Disability in American Culture and Literature* (New York: Columbia University Press). For a wider cultural history of the ‘normal’ that tracks the concept from the 19th century, see Peter Cryle and Elizabeth Stephens, 2017, *Normality: A Critical Genealogy* (Chicago and London: The University of Chicago Press).

2. Michael Davidson, 2008, *Concerto for the Left Hand: Disability and the Defamiliar Body* (Ann Arbor: The University of Michigan Press), 4.

3. Minsoo Kang, 2011, *Sublime Dreams of Living Machines: The Automaton in the European Imagination* (Cambridge, Massachusetts: Harvard University Press), 5 and 12.

4. James Porter, 1997, “Foreword,” in *The Body and Physical Difference: Discourses of Disability*, ed. David Mitchell and Sharon Snyder (Ann Arbor: Michigan), xiii. Italics in original.

5. See, as an example, the essays collected in *The Human Enhancement Debate and Disability: New Bodies for a Better Life*, ed. Miriam Eilers et al., 2014 (Basingstoke: Palgrave Macmillan).

6. Alison Landsberg, 2004, *Prosthetic Memory: The Transformation of American Remembrance in the Age of Mass Culture* (New York: Columbia University Press), 2 and 9.

7. Margrit Shildrick, 2015, “’Why Should Our Bodies End at the Skin’: Embodiment, Boundaries and Somatechnics,” *Hypatia* 30, no. 1: 14. See also Margrit Shildrick (2009), “Prosthetic Performativity: Deleuzian Connections and Queer Corporealities,” in *Deleuze and Queer Theory*, ed. Chrysanthi Nigianni and Merl Storr (Edinburgh: Edinburgh University Press), 115–33; and Margrit Shildrick, 2013, “Re-imagining Embodiment: Prostheses, Supplements and Boundaries,” *Somatechnics* 3, no. 2: 270–286.

8. Cassandra Crawford, 2014, *Phantom Limb: Amputation, Embodiment, and Prosthetic Technology* (New York: New York University Press), 13, 10–11 and 6.

9. Maria Neicu, 2012, “Prosthetics Imagery: Negotiating the Identity of Enhanced Bodies,” *Platform* 6, no. 2 (Summer): 56.

10. Vivian Sobchack, 2006, “A Leg To Stand On: Prosthetics, Metaphor and Materiality,” in *The Prosthetic Impulse: From a Posthuman Present to a Biocultural Future*, ed. Marquand Smith and Joanna Morris (Cambridge, Massachusetts: MIT Press), 21.

11. Sarah S Jain, 1999, “The Prosthetic Imagination: Enabling and Disabling the Prosthesis Trope,” *Science, Technology and Human Values* 24, no. 1, (Winter): 32 and 33.

12. Sherryl Vint, 2007, *Bodies of Tomorrow: Technology, Subjectivity, Science Fiction* (Toronto: University of Toronto Press), 7 and 104.

13. Herbert A Simon, 1996, *The Sciences of the Artificial*, 3rd ed. (Cambridge, Massachusetts: MIT Press).

14. Donald A Schön, 1995, *The Reflective Practitioner: How Professionals Think in Action* (Aldershot, Arena).

15. Louis Bucciarelli, 1994, *Designing Engineers* (Cambridge, Massachusetts and London, MIT Press).

16. Bucciarelli, *Designing Engineers*, 18–19.

17. Graham Pullin, 2009, *Design Meets Disability* (Cambridge, Massachusetts and London, MIT Press), 89–90 and 303.

18. Bucciarelli, *Designing Engineers*, 195.

19. Royal Academy of Engineering, "Creating Systems that Work: Principles of Engineering Systems for the 21st Century,” https://www.raeng.org.uk/publications/reports/rae-systems-report.

20. Royal Academy of Engineering, *"*Creating Systems that Work,” 16.

21. The place and role of users is complex here. Users are often consulted at the start and end of the modelling process: they help to frame the issue, and then are re-engaged to test whether the identified need has been met. Ideally, users should be involved or consulted throughout development to identify issues as they develop, but systems engineering works to compartmentalise the development process as far as possible so that engineers or designers working on different sections do not need to interact with others. The difference between products is also important here: in some cases, there are subsystems where users do not need to be considered at all; and others (eg, user interfaces, wearables or prosthetics—devices connected to the body) where they need to be involved frequently.

22. See Henry Dreyfus, 1967, *The Measure of Man: Human Factors in Design*, 2nd ed. (New York: Whitney Library of Design), and the updated version of Dreyfus’ work: Alvin R. Tilley, *The Measure of Man and Woman: Human Factors in Design* (New York: Wiley, 2002).

23. Patrick W Jordan, 2000, *Designing Pleasurable Products: An Introduction to the New Human Factors* (London: Taylor and Francis).

24. See Konstantin D. Bergmeister et al. (2017), “Broadband Prosthetic Interfaces: Combining Nerve Transfers and Implantable Multichannel EMG Technology to Decode Spinal Motor Neuron Activity,” *Frontiers in Neuroscience* 11, no. 421: https://doi.org/10.3389/fnins.2017.00421.

25. See Tomasz Miaskiewicz and Kenneth A. Kozar, 2011, “Personas and User-Centred Design: How Can Personas Benefit Product Design Processes?,” *Design* *Studies* 32, no. 5: 417–30.

26. John K. Christiansen and Marta Gasparin, 2016, “Managing Controversies in the Fuzzy Front End,” *Creativity and Innovation Management* 25, no. 4: 500–14.

27. Vivian Sobchack, 2006, Leg to Stand On, 27.

28. Manuela Rossini, 2017, “Bodies,” in *The Cambridge Companion to Literature and the Posthuman*, ed. Bruce Clarke and Manuela Rossini (Cambridge: Cambridge University Press), 164

29. Peter Kroes, 2010, “Engineering and the Dual Nature of Technical Artefacts,” *Cambridge Journal of Economics* 34, no. 1: 51–62.

30. Alison Kafer, 2013, *Feminist, Queer, Crip* (Bloomington and Indianapolis: Indiana University Press), 20.

31. Despina Kakoudaki, 2014, *Anatomy of a Robot: Literature, Cinema, and the Cultural Work of Artificial People* (New Brunswick, New Jersey and London: Rutgers University Press), 3, 12 and 14.

32. Louis Bucciarelli, 1994, *Designing Engineers*, 196.

33. Margrit Shildrick, 2015, “Why Should Our Bodies End at the Skin,” 14.

34. William Viney et al., 2015, Felicity Callard, Angela Woods, “Critical Medical Humanities: Embracing Entanglements, Taking Risks,” *Journal of Medical Humanities* 41, no. 1: 2–7.

35. Pramod Nayar, 2014 *Posthumanism* (Cambridge: Polity), 9 and 64.

36. Rosi Braidotti, 2013 *The Posthuman* (Cambridge: Polity), 49.

37. David Mitchell and Sharon Snyder, *Narrative Prosthesis: Disability and the Dependencies of Discourse* (Ann Arbor: University of Michigan Press, 2000), 7.
